# Supplementary material for: Proteotranscriptomic Profiling of the Toxic Mucus of Kulikovia alborostrata (Pilidiophora, Nemertea)
Source: Toxins (Basel). 2024 Dec 26;17(1):5. doi: 10.3390/toxins17010005 (PMC11768458; doi:10.3390/toxins17010005)
Supplement: Supplementary file 1 [file toxins-17-00005-s001.zip › Supplementary File S4.zip!/ORF 076246 LINALB/5646dc0998f06131/ss_report.pdf]

Email	vas9i-kz@mail.ru
Description	putative_toxin_cistein_rich_
Date	Wed Apr 5 07:02:50 BST 2023
Unique Job ID	5646dc0998f06131

Secondary structure and disorder prediction

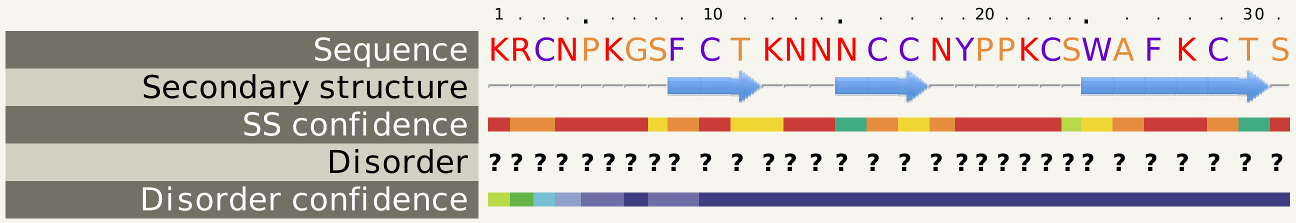

Confidence Key

High(9) Low (0)

? Disordered (29%)

Alpha helix (0%)

Beta strand (39%)
